# Potential Prebiotic Effect of Inulin-Enriched Pasta after In Vitro Gastrointestinal Digestion and Simulated Gut Fermentation

**DOI:** 10.3390/foods13121815

**Published:** 2024-06-08

**Authors:** Anna Rita Bavaro, Mariaelena Di Biase, Vito Linsalata, Isabella D’Antuono, Vita Di Stefano, Stella Lisa Lonigro, Antonella Garbetta, Francesca Valerio, Maria Grazia Melilli, Angela Cardinali

**Affiliations:** 1 Institute of Sciences of Food Productions (ISPA), National Research Council (CNR), 70126 Bari, Italy; annarita.bavaro@ispa.cnr.it (A.R.B.); mariaelena.dibiase@ispa.cnr.it (M.D.B.); vito.linsalata@ispa.cnr.it (V.L.); isabella.dantuono@ispa.cnr.it (I.D.); lisa.lonigro@ispa.cnr.it (S.L.L.); antonella.garbetta@ispa.cnr.it (A.G.); angela.cardinali@ispa.cnr.it (A.C.); 2 Department of Biological, Chemical and Pharmaceutical Sciences and Technologies, University of Palermo, 90123 Palermo, Italy; vita.distefano@community.unipa.it; 3 Institute of Biomolecular Chemistry (ICB), National Research Council (CNR), 95126 Catania, Italy

**Keywords:** *Lacticaseibacillus paracasei* IMPC2.1, SCFA, inulin, pasta, functional food

## Abstract

In the current study, the prebiotic potential of an innovative functional pasta enriched with 12% (*w*/*w*) inulin was investigated. To this aim, pasta was subjected to in vitro gastrointestinal digestion followed by simulated gut fermentation compared to the control pasta (CTRL) not containing inulin. The incorporation of inulin positively (*p* < 0.05) affected some organoleptic traits and the cooking quality of the final product, giving an overall score significantly higher than CTRL. The resultant essential amino acid content was similar in both pasta samples while the total protein content was lower in inulin-enriched pasta for the polymer substitution to durum wheat flour. The prebiotic potential of chicory inulin was preliminarily tested in in vitro experiments using seven probiotic strains and among them *Lacticaseibacillus paracasei* IMPC2.1 was selected for the simulated gut fermentation studies. The positive prebiotic activity score registered with the probiotic strain suggested the suitability of the inulin-enriched pasta with respect to acting as a prebiotic source favoring the growth of the probiotic strain and short chain fatty acid (SCFA) production. The present study contributes to broadening knowledge on the prebiotic efficacy of inulin when incorporated into a complex food matrix.

## 1. Introduction

In order to satisfy the consumer’s request for a healthier diet, the functional food market is constantly investing in research studies leading to the development of innovative functional foods. Functional foods have been defined as “Natural or processed foods that contain biologically active compounds which, in defined, effective and non-toxic amounts, provide a clinically proven and documented health benefit utilizing specific biomarkers for the prevention, management, or treatment of chronic disease or its symptoms” [1]. The food sector is now continuously working to develop innovative functional foods to be included in the daily diet of consumers. Among them, several products fortified with prebiotic substances, known for their beneficial effect on the gut microbiome, have been developed. An imbalance in the microbiota has been linked to several health problems, including autoimmune disorders, cardiovascular disease, certain types of cancer, obesity and other metabolic disorders [2,3,4]. Prebiotics are defined as “a substrate selectively utilized by host microorganisms conferring a health benefit”: they are non-viable food components that are not hydrolyzed or absorbed in the upper part of the gastrointestinal tract but could be selectively used as a carbon source by beneficial microorganisms (e.g., *Lactobacillus* and *Bifidobacterium*) when they reach the large intestine, thus modulating the intestinal microbiota and positively impacting the intestinal barrier functions [5,6,7]. By mimicking intestinal binding sites, some prebiotics inhibit the pathogenic microbiota’s adhesion to the gastrointestinal tract [8]. Prebiotics can also positively affect the immune system by modulating the gut microbiota [9,10], upregulating interleukins and immunoglobulins, downregulating proinflammatory interleukins [11,12] and improving short-chain fatty acid (SCFA) production [13]. SCFAs are absorbed by epithelial cells and used as energy sources and metabolic regulators improving the intestinal barrier integrity acting on villi growth, crypt development, tight junctions and mucin production [14]. Moreover, SCFAs are an essential indicator of bacterial fermentation in the colon and protect against inflammation [15].

Among substances that can be considered as prebiotics, the European Food Safety Agency (EFSA) defined resistant oligosaccharides, fructo-oligosaccharides (FOS), galactooligosaccharides (GOS) and other resistant oligosaccharides, resistant starch and inulin as dietary fibers. GOS and FOS are constituted by 3–9 monomers, while fructans and non-starch polysaccharides are constituted by 10 or more monomers (e.g., cellulose, hemicellulose, gums, pectin, mucilage, inulin, psyllium, β-glucan and resistant starch) [16,17]. Inulin belongs to fructans, consisting of chains of fructose units linked via β (2→1) bonds with a single D-glucosyl unit at the non-reducing end [18,19] and has β-(2,1) linkages between fructofuranosyl units and branching at the β-(2,6) location.

Fructans are selectively fermented in the large intestine by *Bifidobacteria* which are rather sensitive to fructans degree of polymerization (DP) [20]. Inulin represents a noteworthy ingredient widely used in a variety of foods. Its particular structure avoids hydrolyzation by the human enzymes occurring in the gastrointestinal tract [21]. The recommended daily intake of inulin is approximately 20 g/day and no associated adverse reactions suggest that divided doses can be taken multiple times per day. 

Among foods functionalized with prebiotics, pasta represents a valuable alternative since it is a widely and frequently consumed popular food, characterized by low glycemic index. Melilli et al. [22] reviewed the health benefits of different inulin-fortified foods and the applications in the food industry even in relation to the inulin DP. Inulin addition to food products increased their nutritional and health properties, compromising the final taste and consistency; it has been used as a sweetener and as a substitute for fats and carbohydrates, decreasing the glycemic index. Sissons [23] reported an overview of the recent scientific articles published on pasta enriched with functional ingredients. Effects on α-amylase inhibition in relation to the degree of polymerization of inulin added in pasta were also reported [24].

Prebiotics can differently affect the gut microbiota. Yin et al. [9] explored the microbiota dynamic response during the in vitro fermentation of FOS and inulin using two groups of human fecal slurries: bacteroides/bifidobacterium high (H) and bacteroides/bifidobacterium low (L). Authors observed seven co-abundance response groups (CARGs) with CARG 4 being the best predictor of inulin SCFAs since it is positively correlated with acetic acid and negatively with propionic and butyric acids. This result confirms the strong relationship between fructoligosaccharides and gut microbiota composition, reinforcing the trend of tailored functional diets for ameliorating the gut health by modulating the microbiota. The efficacy of inulin in modulating the gut microbiota was also demonstrated by An et al. [25] who observed a significant effect of the simultaneous and pre-supplementation of inulin in mitigating the antibiotic-induced dysbiosis. More recently, a wide range of innovative substances have been suggested as prebiotics, including collagen peptides from different food fonts and can act as a nitrogen or carbon source for gut microbiota and microalga polysaccharides. The consumption of micro- and macroalgae polysaccharidic components has been correlated with the promotion of specific probiotics and suppression of other harmful gut bacteria [26]. The interest in inulin as a functional fiber with potential beneficial effects on human health is mainly related to the DP. This parameter could also differently affect the gut microbiota as demonstrated by Ariaee et al. [27]. The authors assessed that the growth of several beneficial taxa from the *Bifidobacterium* genera was favored by inulin at DP 7, whilst the medium (DP 14) and long-chain inulin (DP 27) induced a unique microbial composition. At the same time, a reduction in total weight gain and systemic glucose levels was observed with long-chain inulin, suggesting a DP-specific effect on metabolic health. Difonzo et al. [28] used inulin from artichoke roots to produce functional fresh pasta and showed that, after in vitro gastrointestinal digestion, the inulin-enriched pasta increased the cell density of probiotic strains that were able to significantly inhibit the growth of *E. coli*. The combination of prebiotics with probiotics could strongly empower the efficacy of the single components modulating the gut microbiota and relevant metabolites such as SCFAs as demonstrated in our previous studies with ready-to-eat artichokes enriched with *Lacticaseibacillus paracasei* IMPC2.1 [29,30,31]. 

The incorporation of a prebiotic compound in the food formulation can have implications on the bioavailability of the fiber in the gut. In designing a functional pasta, consideration must be given to the processing steps (extrusion or lamination process, the drying process, high temperature and humidity) that can cause a loss in efficacy of the active ingredient. 

The growing elderly population and concerns about gut health and muscle mass are fueling demand for products in several applications. Consumer spending on products that improve gut health is expected to increase in the coming period, leading the global prebiotics market to grow 14.9% annually to reach USD 21.2 billion by 2030 [32]. 

The administration of dietary fibers through the diet is a therapeutic strategy recently adopted to treat several diseases and new studies are essential to develop new foods with prebiotic action. Currently, most of the research studies finalized for the assessment of prebiotic features of specific dietary fibers have been performed on purified prebiotic compounds while only a few data are available on the efficacy of inulin incorporated in a complex food matrix. 

In this study, pasta enriched with 12% inulin from chicory was characterized for biochemical and technological properties. Furthermore, in order to investigate the potential prebiotic effect, the enriched pasta was subjected to in vitro gastrointestinal digestion, followed by fermentation with the probiotic strain *Lacticaseibacillus paracasei* IMPC2.1 and the enteric strain *Escherichia coli* ATCC35401. The effect of digested pasta on probiotic growth and metabolism, in terms of SCFA production, was also studied.

## 2. Materials and Methods

### 2.1. Chemicals and Reagents

Methanol, ethanol, pyridine anhydrous, ethyl chloroformate, hydrochloric acid and sodium bicarbonate were purchased from Sigma-Aldrich (St. Louis, MO, USA). 

Fructose (Oxoid, Basingstoke, UK), fructo-oligosaccharides from chicory (Sigma Aldrich) and inulin from chicory (Sigma-Aldrich) were used for in vitro prebiotic potential evaluation.

Porcine pancreas α-amylase (A3176, Sigma-Aldrich), pepsin from porcine gastric mucosa (P7125, Sigma-Aldrich), pancreatin from porcine pancreas (P1750, Sigma-Aldrich), lipase from porcine pancreas (L3126, Sigma-Aldrich) and porcine bile extract porcine (B8631, Sigma-Aldrich) were used for in vitro digestion. 

A mix of 17 Amino acid standards containing L-Ala, L-Arg, L-Asp, L-Glu, L-Gly, L-His, L-Ile, L-Leu L-Lys, L-Met, L-Phe, L-Pro, L-Ser, L-Thr, L-Tyr and L-Val at 0.5 μmole/L and L-Cys at 0.25 μmole/L (Supelco) were used. L-Trp, L-Asn and L-Gln pure standards, hydrochloric acid 37%, formic acid, acetonitrile, methanol, acetone, boric acid, sodium hydroxide, calcium carbonate, heptylamine and 9-Fluorenylmethoxycarbonyl chloride (FMOC-Cl) were acquired from Merck (Darmstadt, Germany). HPLC-grade water was produced by purifying double distilled water in a Milli-Q Gradient A10 system (Millipore SAS, Molsheim, France).

### 2.2. Inulin-Enriched Pasta

Inulin-enriched pasta was produced with Senatore Cappelli (CAP) durum wheat semi whole-meal flour, adding 12% (*w*/*w*) commercial chicory inulin. Senatore Cappelli semolina proximate composition was carbohydrates (66.3%), proteins (12.7%), fiber (7.7%) and fat (2.1%), while commercial inulin was provided by ORAFTI (Tienen, Belgium) as Raftilose^®^ Synergy 1, a mixture 1/1 of long-chain (DP ranging from 10 to 65, average 25) and short-chain (DP ranging from 3 to 8, average 4) fractions of inulin extracted from chicory roots (*Cichorium intybus*). 

To produce pasta, inulin was previously dissolved in water and then was mixed with semolina. Spaghetti (30 cm in length × 1.70 mm) were extruded using the operating conditions described by Melilli et al. [24]. Pasta without inulin was used as control (CTRL). 

On both types of spaghetti, sensory attributes and cooking quality parameters were determined, as described by Di Stefano et al. [33]. The panel group had six men and nine women, aged between 28 and 65, and a nine-point scale (1 extremely unpleasant, 9 extremely pleasant and 5 at the threshold of acceptability) was used to quantify each attribute [34]. Based on the above-mentioned attributes, panelists were also asked to score the overall quality (OQS) of the product using the same scale.

The optimal cooking time (OCT) was evaluated according to the AACC-approved method 66-50 (2000). The swelling index of cooked pasta was determined according to the procedure described by Cleary and Brennan [35]. The measurements were performed in triplicates for each analysis.

Pasta color was measured in spaghetti before and after cooking at OCT using a Chroma Meter (Minolta CR—400, Milan, Lombardy, Italy) [24].

**Figure 1:**
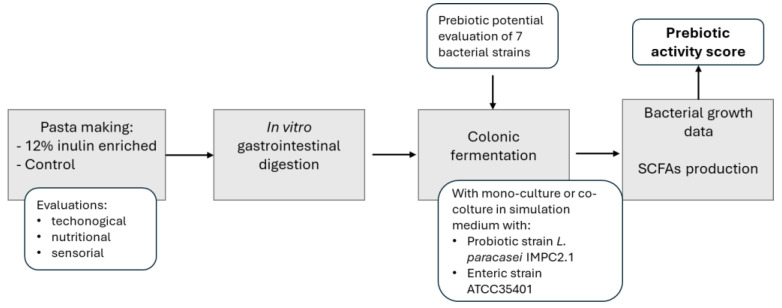
Flowchart of the study design.

Inulin-enriched pasta was then subjected to different analyses to ascertain its technological and nutritional quality and to in vitro gastrointestinal digestion followed by simulated gut fermentation, as reported in Figure 1.

### 2.3. Determination of Total Protein Content and Amino Acid Content

Total protein content was determined using Kjeldahl’s method [36]. In Senatore Cappelli (CAP) durum wheat pasta (CTRL), and in pasta enriched with 12% chicory inulin, the amino acid content was investigated using a modified procedure employing acid hydrolysis of protein. Subsequent precolumn derivatization of the free amino acids was performed using 9-fluorenylmethyl chloroformate (FMOC-Cl) [37]. The separation of the derivatized amino acids was performed on reversed-phase HPLC columns and the quantification was performed based on their fluorescent properties. Briefly, the sample was reacted in a clean hydrolysis tube with 6 M HCl at 110–120 °C for 24 h. After cooling, the sample was evaporated to dryness at 60 °C. The residue was then dissolved in 2 mL of high-purity water and filtered through 0.45 µm PTFE syringe filters. The mixture containing the amino acids in free form was then derivatized at room temperature with FMOC-Cl. After the addition of borate buffer solutions and FMOC-Cl, the solution was heated at 70 °C for 10 min. Excess FMOC-Cl was removed with heptylamine solution. Subsequently, the solution was injected into the HPLC-FLD to obtain chromatographic amino acid fingerprints. Chromatographic separation was performed with a Discovery HS C18 analytical column under gradient elution conditions using 0.1% formic acid solution (solvent A) and acetonitrile (solvent B) as mobile phase components. The mobile phase flow rate was 1 mL/min. Amino acid chromatographic separation was recorded at an excitation wavelength of 254 nm and an emission wavelength of 630 nm. For peak identification, the comparison between the retention times of the amino acid standards and samples was carried out applying a fortification technique (spiking). For quantitation, an external standard method using calibration curves fitted by linear regression analysis was utilized. The results were expressed in grams of amino acids per 100 g of product. Standard mix solutions of amino acids were prepared in concentrations from 0.1 μmole/L to 1 μmole/L and from 0.25 μmole/L to 1.87 μmole/L for L-Trp, L-Asn and L-Gln and analyzed in triplicate. Linearity was determined by the least square regression (R^2^). Amino acid derivatives showed good linearity under the proposed conditions. Total Amino Acids (TAAs) were expressed in g/100 g of sample.

### 2.4. Microorganisms and Growth Conditions for Preliminary Prebiotic Potential Evaluation

Microorganisms used for the preliminary study of prebiotic potential of chicory inulin were obtained both from the CNR-ISPA Collection and from Chr. Hansen (Hørsholm, Denmark). Strains from CNR ISPA were the followng: *Lacticaseibacillus casei* IMPC2.1, IMPC4.1 and *Lacticaseibacillus paracasei/casei* P1. The remaining strains *Bifidobacterium animalis* ssp. *lactis* Bb12, *Lacticaseibacillus casei* 01, *Lacticaseibacillus casei* 431 and *Lacticaseibacillus rhamnosus* GG were provided by Chr. Hansen (Hørsholm, Denmark). All strains provided by Chr. Hansen were classified as probiotics according to their manufacturers. The probiotic strain *Lacticaseibacillus paracasei* IMPC2.1 (from human intestine) was shown to have functional properties, i.e., immunomodulatory, anti-proliferative and pro-apoptotic effects [38,39,40]. Strain *Lactocaseibacillus paracasei* IMPC4.1 (from human intestine) has been deeply studied for its potential probiotic features [40]. Finally, strain P1 was isolated from fermented table olives [41] and subsequently identified as *L. paracasei*/*casei* by 16S rRNA gene sequence analysis. Strains provided by Chr. Hansen were already tested in a research study aimed to determine the prebiotic potential of an insect flour in in vitro gut microbiota model de Carvalho et al. [42].

For the simulated gut fermentation experiments, strain *L. paracasei* IMPC2.1 was used in combination with the enteric pathogen *Escherichia coli* ATCC35401.

For long-term storage, stock cultures were prepared by mixing 8 mL of a culture in de Man Rogosa Sharpe (MRS) broth (Condalab, Madrid, Spain) for LAB strains or Nutrient Broth (Biolife italiana, Milan, Italy) for the *E. coli* strain, with 2 mL of Bacto glycerol (Difco, Becton Dickinson, Co., Sparks, MD, USA) and freezing 1 mL portions of this mixture at −80 °C. To obtain fresh cultures, the strains were subcultured twice (1% *v*/*v*) for 24 h before use in experiments. Growth conditions used for strains are reported in Table 1.

### 2.5. Preliminary Prebiotic Potential Evaluation

To evaluate the prebiotic potential of chicory inulin, growth curves of the probiotic strains in modified MRS containing chicory inulin as carbohydrate source were determined by turbidity measurement using Bioscreen C (Oy Growth Curves Ab Ltd., Helsinki, Finland) following the protocol used in Jiménez-Sánchez et al. [43] with slight modifications. A fresh overnight culture of each strain was prepared in MRS broth and MRS broth supplemented with 0.5 g/L cysteine hydrochloride for *B. animalis* subsp. *lactis* Bb12. Cells from a culture with an optical density at 600 nm of 0.1 were harvested by centrifugation at 10,416× *g* (10,000 × rpm in a rotor 19777, 10 min, 4 °C, centrifuge 3K30, Sigma Laboratory Centrifuge, Osterode, Germany), washed three times in freshy prepared sterile phosphate buffered saline (PBS, pH 7.2), supplemented with 0.5 g/L cysteine hydrochloride (Sigma-Aldrich) and resuspended in MRS-medium without sugar (bactopeptone 10 g (Conda, Madrid, Spain), beef extract 8 g (Biolife italiana, Milan, Italy), yeast extract 4 g (Biolife italiana), di-potassium hydrogen phosphate 2 g (VWR Chemicals BDH Prolabo, Australia), tween 80 2 g (VWR Chemicals BDH Prolabo), ammonium citrate tribasic 2 g (Sigma-Aldrich), sodium acetate 5 g (Riedel-deHaen, Seelze, Germany), anhydrous magnesium sulphate 0.2 g (Polichimica, Bologna, Italy), manganese sulphate 0.04 g (Polichimica)) in the same initial volume. 

The following media were inoculated in triplicate in the honeycomb plate to obtain a final microbial load of 10^6^ CFU/well: negative control medium not containing a sugar source, MRS broth enriched with 1% of a carbohydrate source as fructose (FR, Oxoid), fructo-oligosaccharides from chicory (FOS, Sigma-Aldrich) or inulin from chicory (Inc, Sigma-Aldrich). Plates were incubated in the Bioscreen C at 37 °C for at least 48 h and the OD600 was measured every hour after mixing for 15 s. The growth curves were determined in three replicates and the results are shown as a mean. The growth induction period, duration of time until reaching the exponential multiplication phase of the bacteria and corresponding to the lag phase, as well as the maximum optical density (600 nm) were determined as reported in Kneifel [44] and Jiménez-Sánchez et al. [43]. Briefly, the growth induction period was calculated with the turbidimetric data considering the time needed to start the exponential growth phase. The maximum optical density was determined considering the incubation time showing the highest OD value during the stationary phase. For this calculation, a period of at least 10 h showing no modification of the OD value was considered.

### 2.6. In Vitro Gastrointestinal Digestion

In vitro gastrointestinal digestion of pasta enriched with 12% inulin from chicory and control pasta (CTRL) was performed following the method of Garbetta et al. [45]. The samples of enriched and CTRL pasta were cooked to their optimum cooking time (OCT) and then 6 g was subjected to in vitro digestion process. Briefly, the procedure reproduces the physiological three steps of the digestive process: oral (10 min), gastric (1 h) and small intestine (2 h). The oral stock solution and the gastric and intestinal enzyme solutions were prepared as reported by D’Antuono et al. [46]; α-amylase and pepsin were used in the oral and gastric phase, respectively; pancreatin, lipase and bile extract were used in the intestinal step. 

At the end of the in vitro digestion, an aliquot of samples was centrifuged at 8437× *g* (9000 rpm) for 10 min, obtaining the aqueous small intestinal digesta (DG) and solid fraction pellet (PT) utilized for inulin content analysis. The remaining parts of digesta samples were transferred to 1 kDa nominal molecular weight cut-off regenerated cellulose dialysis tubing (Spectra/Por^®^ 6, Spectrum Europe BV, Breda, The Netherlands) and dialyzed against 0.01 M NaCl at 5 ± 0.5 °C for 15 h, followed by further 2 h after replacing the dialysis solution, as reported in de Albuquerque et al. [47]. All the enzymes and reagents used to simulate the gastrointestinal digestion were purchased from Sigma-Aldrich. The dialyzed digested samples were freeze-dried and stored at 5 ± 0.5 °C for a maximum of 4 weeks. The digested pasta samples (inulin-enriched and CTRL) were assayed individually as a single carbon source, in comparison to glucose and FOS. Each experiment was carried out in triplicate.

### 2.7. Inulin Quantification

The glucose, fructose and sucrose analysis and quantification of inulin as total fructose after acid hydrolysis were performed on the samples before and after in vitro digestion following the method reported by Garbetta et al. [45] with slight modifications. Briefly, the inulin extraction was carried out on the cooked pasta at the established OCT in 50 mL of boiling water at 100 °C for 30 min and then centrifuged at 3000× *g* (3946 rpm in a rotor SX4250, Allegra X-22R centrifuge, Beckman Coulter, Palo Alto, CA, USA) at room temperature (20 to 25 °C) for 15 min. An aliquot of recovered supernatant was filtered at 0.45 μm and analyzed in HPLC for the quantification of free sugars. At the same time, another fraction underwent acid hydrolysis with 0.03 N HCl for 1 h at 70 °C; afterwards, it was cooled, filtered and analyzed in HPLC for inulin quantification. To verify the absence of inulin and sugars that might interfere with quantification, an extraction process was also performed on the raw CTRL pasta. The HPLC DX500 (Dionex, Sunnyvale, CA, USA) system equipped with a GP50 gradient pump, ED40 Electrochemical Detector in Pulsed Amperometric Detection (PAD) and DionexPeaknet 5.11 chromatographic Software was used for carbohydrate analysis. For the sugars’ chromatographic separation, a Dionex CarboPac PA1 column and Carbopac PA1 guard column in isocratic mode with elution of 150 mM NaOH at flow rate of 1 mL/min were applied. 

The digested samples (DG and PT fractions) were directly analyzed for the free sugar quantification and extracted following the procedure for inulin analysis described above.

For the calculation of the amount of fructose from inulin, the following formula was applied:

F_i_ = F_t_ − F_f_ − F_s_ (1)

where F_i_ = fructose from inulin, F_t_ = total fructose after hydrolysis, F_f_ = fructose free and F_s_ = fructose from sucrose before hydrolysis. Inulin content (I) was calculated according to the procedure described by Steegmans, Iliaens and Hoebregs [48]:

I = 0.995 F_i_ (2)

The cooking loss of inulin was determined after cooking the enriched pasta at its OCT and residual cooking water was collected and analyzed for the inulin content by HPLC as previously described. Results were expressed as grams of inulin lost in the cooking water from 100 g of pasta. The analysis was carried out in triplicate.

### 2.8. Simulated Gut Fermentation 

After in vitro digestion, to assess the ability of the digested inulin-enriched pasta to stimulate the growth and the metabolic activity of the probiotic strain *L. paracasei* IMPC2.1 in the presence or not of the enteric pathogen *Escherichia coli* ATCC35401, a fermentation experiment in gut simulation medium containing digested inulin-enriched pasta or glucose or FOS as carbohydrate source in comparison to digested CTRL pasta (not containing inulin) was performed. The probiotic features of the *L. paracasei* strain have been highlighted in clinical studies [29] and human feeding trials [30], using vegetable or fish matrices as a carrier. The inoculum of strains was prepared as reported in de Albuquerque et al. [47] with slight modification. Briefly, the selected strains *L. paracasei* IMPC2.1 and *E. coli* ATCC35401 were cultivated two times in MRS broth or Nutrient Broth, respectively, at 37 °C for 8 h, and subcultured in fresh medium. After overnight growth, the cultures were centrifuged at 4500× *g* at 4 °C for 15 min, washed two times and re-suspended in sterile saline solution (NaCl 8.5 g/L) to obtain a cell suspension with an optical density at 655 nm of 0.8 (*L. paracasei*) or 0.1 (*E. coli*). This suspension provided viable cell counts of ~8 log colony forming units (CFU)/mL when plated on MRS agar.

The inoculum (0.1 mL) was placed in sterile flasks containing 5 mL of gut bacterial simulation growth media, prepared according to Madureira et al. [49]. The composition of this media was 5.0 g/L trypticase soy broth (TSB) without dextrose (Biolife Italiana) 5.0 g/L bactopeptone (Conda), 5.0 g/L yeast nitrogen base (Biolife Italiana), 1.0% (*v*/*v*) of salt solution A (100.0 g/L NH_4_Cl, 10.0 g/L MgCl_2_·6H_2_O and 10.0 g/L CaCl_2_·2H_2_O), 0.2% (*v*/*v*) of salt solution B (200.0 g/L K_2_HPO_4_·3H_2_O), 0.2% (*v*/*v*) of 0.5 g/L resazurin solution and 10.0 mL/L trace mineral supplement (ATCC, Manassas, VA, USA) in distilled water.

The gut simulation medium was used as basal broth with glucose (20 g/L), FOS (20 g/L), digested CTRL pasta (20 g/L) or inulin-enriched pasta (20 g/L). The selected probiotic or enteric strains were used as mono-culture or co-culture. Digested pasta or FOS or fructose were added to the simulation media at 2% (*w*/*v*) and the bacterial strains were inoculated at 2%. The simulation of fermentation was performed for 48 h at 37 °C under anaerobic conditions. To calculate the prebiotic activity score in the mono-cultures with respect to glucose as a carbon source, the equation reported in de Albuquerque et al. [47] was used:

Prebiotic activity score in the mono-culture = [(prob. log CFU/mL on the preb. at 48 h − prob. log CFU/mL on the preb. at 0 h)/(prob. log CFU/mL on glucose at 48 h − prob. log CFU/mL on glucose at 0 h)] − [(enteric log CFU/mL on the preb. at 48 h − enteric log CFU/mL on preb. at 0 h)/(enteric log CFU/mL on glucose at 48 h − enteric log CFU/mL on glucose at 0 h)].

To calculate the prebiotic activity score in the co-cultures for each carbon source, the equation reported in Kaewarsar et al. [50] was used:

Prebiotic activity score in the co-culture = (prob. log CFU/mL at 48 h/prob. log CFU/mL at 0 h) − (enteric log CFU/mL at 48 h/enteric log CFU/mL at 0 h)/Total ((prob. log CFU/mL at 48 h/prob. log CFU/mL at 0 h) + (enteric log CFU/mL at 48 h/enteric log CFU/mL at 0 h)).

After incubation, an aliquot was used for plating on MRS or TBX agar plates (Conda) to check the strain growth, and the remaining sample, after removal of cells by centrifugation and filter sterilization of the supernatant, was used for SCFA analysis, as detailed below.

### 2.9. Microbiological Analysis after Simulated Gut Fermentation

Just after homogenization (T0) and at 48 h of incubation, each mixture (100 μL aliquots) was serially diluted in sterile saline solution containing tween 80 (0.025%, *w*/*v*), and, subsequently, each dilution (100 μL) was plated on MRS and TBX agar. Plates were incubated at 37 °C for 24–48 h to enumerate the viable cells (log CFU/mL).

### 2.10. SCFA Analysis

After fermentation, each sample was processed for SCFA analysis. In detail, the fermented samples were centrifuged at 4500× *g*, 15 min, 4 °C and the supernatant fluid was treated as reported in Valerio et al. [31] with slight modifications. Briefly, 1 mL samples were thawed and homogenized for 2 min in 9 mL of 0.15 mmol/L H_2_SO_4_ in Milli-Q-purified water using a vortex (Seward, London, UK). A 5 mL portion of the homogenate was centrifuged at 9000× *g* at 2 °C for 20 min and the supernatant was filtered using a microconcentrator (Amicon Ultra-4 Centrifugal Filter Devices, Merck Millipore Ltd., Cork, IRL) with a molecular mass cut-off of 3000 Da (7000× *g*, 20 °C, 40 min). The resulting fluid was 0.22 µm filtered (Millipore, Bedford, MA, USA). An HPLC system (AKTABasic10, P-900 series pump; Amersham Biosciences AB, Uppsala, Sweden) equipped with a three-channel UV detector (Amersham Biosciences 900) set at 210 nm was used to separate SCFAs on a Rezex ROA-organic acid H+ (8%) column (7.80 × 300 mm; Phenomenex, Torrance, CA, USA). The mobile phase was H_2_SO_4_ (0.007 mol/L) (Fluka, Deisenhofen, Germany) pumped at a flow rate of 0.6 mL/min through the column heated to 70 °C. The concentration of SCFA was determined by integrating calibration curves obtained from standards and expressed as mmol/L of digested sample suspension. Quantification limits (LOQs) of acetic, propionic, butyric and valeric were 1.825, 0.147, 0.380 and 0.463 mmol/L, respectively.

### 2.11. Statistical Analysis

All data are presented as mean values ± standard error of the mean determined in triplicate. To evaluate the effect of carbon source on the bacterial growth in the preliminary prebiotic test, data (OD600 values) were analyzed by one-way ANOVA followed by the Tukey HSD. Data were compared between strains and between sugars. In the case of simulated gut fermentation test, growth (CFU/mL) and SCFA (mMol/L), data were analyzed by one-way ANOVA followed by LSD Fisher test. Results were considered as statistically significant when the *p*-value was less than 0.05.

## 3. Results and Discussion

### 3.1. Quality of Pasta

The organoleptic characteristic of inulin-enriched pasta (Table 2) strongly influenced this product affecting consumer acceptability. The threshold of acceptability was set at 5 and the inulin fortified pasta was appreciated by the panelists. The addition of 12% inulin mainly influenced the firmness and the bulkiness so that these attributes were more appreciated by panelists in CTRL samples. The other sensory attributes, i.e., adhesiveness, color, odor, taste and OQS, resulted less influence. 

The conspicuous inulin addition influenced the color indices of cooked pasta: a higher value of L* (66) than CTRL (55) was registered, while a* (red index) and b* (yellow index) were lower.

In general, the OCT and the swelling index decreased with the increase in the percentage of substitution, while the water absorption increased. The polymer addition had a competitive activity for water with starch during pasta formation, and the obtained results for cooking quality confirmed data from previous studies [22,45].

It is known that the quality and structure of wheat products are significantly affected not only by their gluten content but also by gluten composition. Since inulin is a dietary fiber, its addition to durum wheat flour products could affect the quality of the final products. 

Brennan and Tudorica [51] reported that cooked pastas, containing 10% chicory inulin, appeared to have similar internal structures compared to CTRL pasta: a gluten and dietary fiber matrix surrounded starch granules presenting a well-maintained shape due to limited water availability. Also, Manno et al. [52] reported a low number or a weak starch–protein binding when 10 or 15% inulin is added to fortify pasta. The results presented here highlight a decrease in the total protein content, determined by Kjeldhal’s method, mainly due to the polymer substitution for durum wheat flour with values of 8.2 (CTRL pasta) and 7.8 g/100 g (inulin-enriched pasta), while the content of essential amino acids was similar in both types of pasta samples (Table 3). 

When formulating an innovative functional food, it should be taken into account that even if the addition of bioactive compounds can be simple, the challenge is to maintain the functionality of the ingredient and the sensorial and technological properties of foods. Garbetta et al. [45] produced spaghetti enriched with 4% inulin at two different degrees of polymerization (DP) from cardoon roots (high DP) and chicory (low DP), evaluating sensory properties, glycemic index (GI) and the influence of the polymerization degree on the release of inulin after simulated in vitro gastrointestinal digestion. Results demonstrated that DP significantly affected the inulin release after in vitro GI digestion since a higher amount of high DP inulin was recovered in solid fraction of digested spaghetti with respect to low DP inulin. The high polymerization degree inulin did not modify the technological and sensorial properties of spaghetti, allowing their consumption as a functional food while the low DP inulin was less retained during cooking, determining worse sensorial and technological traits with respect to the CTRL. Moreover, it was observed that the higher polymerization degree influenced the inulin release in the digestive tract, supporting the potential prebiotic effect. In the current study, the use of a higher inulin amount (12%) allowed one to obtain an overall quality score higher than CTRL.

### 3.2. Prebiotic Potential of the Chicory Inulin

**Figure 2:**
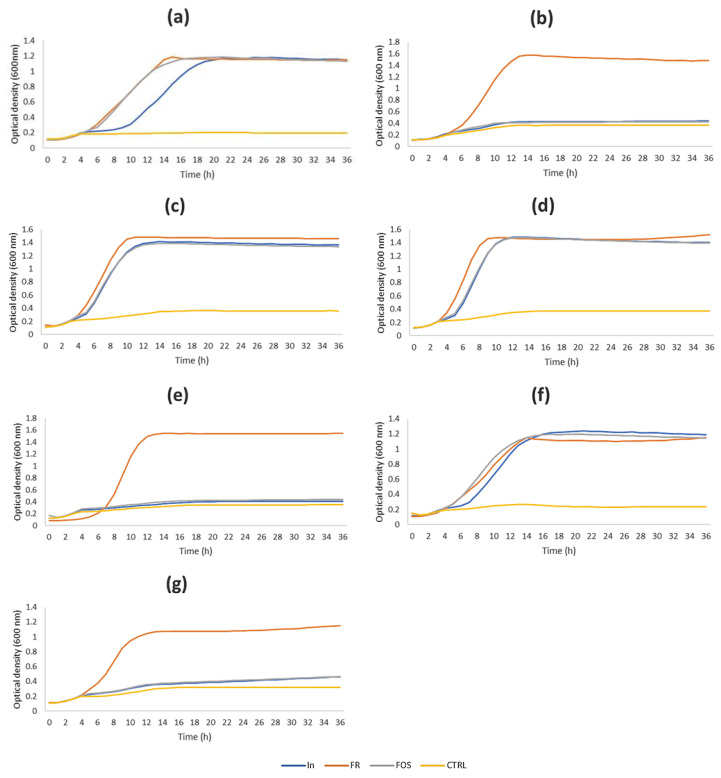
Growth kinetics of *L. casei* 431 (**a**) and 01 (**b**), *L. paracasei* IMPC2.1 (**c**) and IMPC4.1 (**d**), *L. rhamnosus* GG (**e**), *B. animalis* subsp. *lactis* Bb12 (**f**) and *L. paracasei/casei* strain P1 (**g**), without sugars (negative control, yellow line), or in the presence of 1% fructose (FR, orange line) or fructoligosaccharides (FOS, grey line) or chicory inulin (In, blue line) as monitored by the Bioscreen C. The induction period (period until onset of the exponential growth phase used) was calculated as a measure for the duration of the lag phase; the bacterial growth was monitored up to 48 h even if the graph shows the first 36 h, since OD remained stable until the end of experimental period.

To select the most suitable probiotic strains for the evaluation of the prebiotic potential of inulin-enriched pasta after in vitro static GI digestion, the chicory inulin was used as a carbohydrate source in growth experiments. In Figure 2, the growth curves obtained by growing the probiotic strains in MRS medium containing or not containing a carbohydrate source are shown. 

The bacterial growth was monitored up to 48 h, although the first 36 h were enough to calculate the maximum OD values for all strains since no modification of OD values was observed for at least 10 h (*p* > 0.05). For all strains, no growth was observed in the CTRL medium with no carbon source. In accordance with Kneifel [44], results about *Bifidobacterium animalis* subsp. *lactis* Bb12 growth demonstrated that the strain had the potential to increase its population in the human intestine utilizing fructose, FOS and inulin. Also, strains *L. casei* 431, *L. paracasei* IMPC2.1 and IMPC4.1 showed growth in the presence of FOS and chicory inulin similar to that observed with fructose. However, the *L. casei* 431 growth curve in the presence of inulin did not have the same shape as depicted for fructose and FOS, due to a longer lag phase as observed in Figure 2. Results from *L. casei* 01, *L. rhamnosus* GG and *L. paracasei/casei* P1 strains indicated that they were able to use only fructose as a carbon source. The growth curves were more deeply analyzed as reported by Kneifel [44] and Jiménez-Sánches et al. [43]. OD values were used to calculate the induction periods (measured for the duration of the lag phase) and the maximum optical density (600 nm) reached at the end of the exponential phase (Table 4).

Strains showed different growth performances depending on the carbon source as observed by the maximum OD values in Table 4. *L. paracasei* IMPC4.1 showed a higher maximum OD in MRS enriched with inulin or FOS with respect to the other strains, followed by strain IMPC2.1, *B. animalis* subsp. *lactis* Bb12 and *L. casei* 431. Strains *L. casei* 01 and *L. rhamnosus* GG grew better in the presence of fructose, reaching the highest maximum OD followed by *L. paracasei* strains, *L. casei* 431, *B. animalis* subsp. *lactis* Bb12 and *L. paracasei/casei* P1. All strains were able to grow in the presence of fructose, although with some differences in the induction periods and maximum OD (Table 4). Interestingly, the maximum OD values registered for *L. casei* 431 in the presence of all sugars until 36 h incubation were comparable (*p* > 0.05) even if reached after different times (different induction periods). The *L. paracasei* IMPC4.1 reached maximum OD in the presence of inulin and values were comparable (*p* > 0.05) to the fructose and FOS. *B. animalis* subsp. *lactis* Bb12 showed higher (*p* < 0.05) maximum OD in the presence of inulin with respect to fructose. *L. paracasei* IMPC2.1 grew at a higher extent in the presence of fructose even if it reached a maximum OD slightly lower (*p* < 0.05) in inulin and FOS. *L. casei* 01, *L. rhamnosus* GG and strain *L. paracasei/casei* P1 grew only in the presence of fructose.

In Table 4, the induction periods are reported. As expected, the induction period for strains in the control medium was not calculated since no growth was observed. Data suggested that the induction period of *L. casei* 431 was longer in the presence of inulin with respect to the other sugars. The *B. animalis* subsp. *lactis* Bb12 growth curve showed a behavior similar to strain 431 mainly in the lag phase. For both strains, fructose was the preferred carbohydrate source supporting their growth. The two *L. paracasei* strains IMPC2.1 and IMPC4.1 showed the same induction periods in the presence of each carbon source. 

The prebiotic character of chicory inulin by in vitro test has been previously reported [53] and results indicated a growth of lactobacilli and bifidobacteria significantly promoted by inulin fractions at low DP (in particular, inulin fraction with DP 2) although with a growth delay compared to fructose. In our study, results indicated that only four strains were able to grow in the presence of inulin, with different growth behaviors and *L. paracasei* strains reached the highest maximum OD values with almost lower induction periods. Although the two *L. paracasei* strains behaved very similarly, IMPC2.1 was selected as the best candidate for the simulated gut fermentation studies since its well-known probiotic properties were also derived from the in vivo human trials performed after administration of ready-to-eat probiotic artichokes, an inulin-rich food matrix [29,31]. 

### 3.3. Inulin Quantification after In Vitro GI Digestion of Inulin-Enriched Pasta 

The cooking loss of pasta enriched with 12% inulin from chicory roots was about 30%. This result was different from the results reported by Garbetta et al. [45], where a maximum of 7.54% inulin loss was reported. The possible explanation could be due to the different percentages of inulin used in this study, 12% and 4%, and could be attributable to the effect of the fiber which, during cooking, disrupts the protein matrix with starch leaching and there is a consequent increase in the loss of the component. This behavior is much more evident for inulin at low DP as chicory roots as reported by Roberfroid [54] and Foschia et al. [55]. In addition, although there is a higher cooking loss, the pasta enriched with 12% inulin preserved two times more inulin with respect to the pasta enriched with 4% that, as reported successively, could be more suitable to exert a prebiotic effect.

The sugar quantification performed on CTRL pasta showed a low amount of fructose since it was almost completely lost during the cooking procedure.

In order to evaluate the potential interaction with some selected microorganisms simulating a prebiotic effect, the cooked pasta enriched with 12% inulin from chicory was submitted to an in vitro gastrointestinal digestion. In particular, about 85 mg of inulin per gram of raw pasta corresponding to 30 mg per gram of cooked pasta was digested.

The inulin was quantified on the aqueous and solid fractions (DG and PT) of digested pasta. The results obtained demonstrated the presence of inulin in both DG and PT of about 60% and 12%, respectively, confirming the total 70% inulin present in cooked pasta. The results obtained are in agreement with those reported by Garbetta et al. [45], where the release of low DP inulin, as chicory, was higher in the aqueous fraction with respect to the amount of inulin recovered in the pellet. Considering that inulin is a non-digestible carbohydrate, to improve the amount of soluble fiber available for fermentation of the colonic micro-flora, the two fractions were combined. Numerous published papers report that inulin, due to its chemical configuration, is not hydrolyzed by the characteristic enzymes of the human small intestine and a large part of its quantity reaches the colon, favoring the action of beneficial bacteria producing SCFAs, and permitting one to define inulin as a prebiotic dietary fiber [56,57]. 

### 3.4. Prebiotic Potential of Inulin-Enriched Pasta 

The probiotic strain showed its ability to grow on digested inulin-enriched pasta at a higher extent than in the presence of glucose or FOS, and in particular with respect to CTRL pasta (*p* < 0.05) (Table 5). 

Actually, the level of *L. paracasei* IMPC2.1 in CTRL pasta remained unvaried after fermentation (*p* > 0.05). Results indicated that the inulin contained in 6 g of the digested pasta, corresponding to about 180 mg, was even available for microbial growth after in vitro digestion. The enteric strain *E. coli* ATCC35401 showed a consistent growth in all conditions (*p* < 0.05). In order to evaluate the prebiotic potential of the inulin-enriched pasta, the prebiotic activity score was calculated both in the mono-culture and in the co-culture. Results highlighted some differences in the microbial behavior. The probiotic strain growth was more stimulated by inulin in the mono-culture with respect to the co-culture and the relevant prebiotic activity score for inulin-enriched pasta was significantly higher, suggesting that both microorganisms competed for the carbon sources. Actually, the *E. coli* strain growth was favored when cultivated alone compared to in the presence of the *L. paracasei* strain. In particular, the prebiotic activity score calculated individually on each bacterial strain with respect to the growth in the presence of glucose revealed the suitability of the inulin-enriched pasta to act as a prebiotic source, favoring the growth of the probiotic strain *L. paracasei* at the same extent of FOS (*p* > 0.05), while the CTRL pasta favored the growth of the enteric pathogen. When the probiotic strain and the enteric pathogen were inoculated as co-culture, a significant growth of strain IMPC2.1 was observed after fermentation in the presence of FOS or glucose, while the bacterial density remained almost unvaried in inulin-enriched pasta and CTRL pasta. The *E. coli* strain showed a significant increase at T48 in CTRL pasta. However, the prebiotic activity score, calculated in the co-culture for each carbon source, confirmed a positive value for the inulin-enriched pasta and a negative value in CTRL. When pure prebiotic compounds are used to stimulate the growth of beneficial bacteria, different results can be registered, as reported in Kaewarsar et al. [50] who observed a significantly higher prebiotic index after 48 h fermentation for inulin with respect to the glucose in a co-culture containing *Lacticaseibacillus rhamnsosus* and *Bifidobacterium animalis* subsp. *lactis*. In our study, the better probiotic scores were registered for glucose and FOS. This result could be related to the lower availability of the prebiotic partially linked to the food matrix. In addition, different microbial species occurring in the intestinal tract can take part in the fructans metabolism, giving a different result with respect to the in vitro studies. Actually, when the IMPC2.1 strain was administered together with an inulin-rich food matrix such as artichokes, it survived during the GI transit and colonized the intestinal tract being recovered in the fecal samples of the volunteers enrolled for the clinical trials [29]. Results suggested the synergy of action between inulin and the probiotic strain that was able to grow in the colon also favoring SCFA production. Moreover, in vivo studies demonstrated that the IMPC2.1 strain was able to modulate the intestinal microbiota favoring the growth of *Lactobacillus* species, increasing the genetic diversity of lactic population while hampering the proliferation of potentially harmful bacteria, particularly *Escherichia coli* and *Clostridium* spp., when administered to human subjects [29,31]. The lack of an antagonistic activity observed in the current in vitro study could be due to many aspects such as the microbiota composition and the consequent metabolism and the human physiological status. Furthermore, Difonzo et al. [28] used inulin from artichoke roots to produce functional fresh pasta and showed that, after in vitro GI digestion, the inulin-enriched pasta increased the cell density of probiotic strains that were able to significantly inhibit the growth of *E. coli*. Differently from our study, authors used fecal slurries as medium; therefore, a synergistic action in the antagonistic effect could be supposed. 

Inulin also has an important effect on the promotion of the intestinal function barrier integrity [58,59,60,61], an effect particularly evident for inulin at low DP that is more protective for the intestinal barrier integrity with respect to inulin at high DP, as reported by other authors [62,63]. Studies performed on fermented chicory roots containing 75% inulin demonstrated a significant improvement in intestinal barrier integrity [64].

Inulin also has a potential effect in the prevention of non-communicable diseases such as obesity, diabetes, colon cancer, constipation and depression and has been demonstrated to improve its therapeutic effects when added to natural substances showing a possible synergistic effect [21]. So far, experiments conducted with inulin and lactic acid bacteria as synbiotics showed a better role in exerting antidiabetic and antioxidant properties compared with lactic acid bacteria or inulin alone. Many of these health effects are related to the concomitant increase of SCFAs [21].

After the fermentation experiment, the concentration of SCFA was also registered (Table 6). 

Results indicated there is higher lactate production in the presence of glucose followed by FOS. Interestingly, inulin contained in the digested enriched pasta was used by the probiotic strain and converted into lactate, acetate, valerate and propionate, while no butyrate was found in samples in the conditions used for the current study. This result can be related to the very low amount of butyrate produced that was not detected in the experimental conditions. However, the probiotic strain was able to produce butyrate in the presence of FOS and glucose. Butyrate production was observed in the mono-culture *E. coli* in all samples, while a lower lactate production was registered in the co-culture. When strains were cultivated in co-culture, all SCFAs monitored were produced in the presence of digested inulin-enriched pasta. 

SCFAs are currently used as supplements for treating diseases since their decrease is associated with several diseases such as inflammatory bowel disease, irritable bowel disease, mental disorders and dysbiosis. SCFAs have a beneficial role in human health both by improving the intestinal barrier integrity, maintaining immunological and gastrointestinal homeostasis, and by acting as mediators for the gut–brain axis [65]. Each acid is correlated with specific health functions: propionate is mainly correlated with the stimulation of satiety, cholesterol-lowering and antilipogenic effects, and protection against colorectal cancer; acetate is considered a compound required for growth of some bacteria acetate consumers, such as *Faecalibacterium prausnitzii* and *Roseburia intestinalis*/*Eubacterium rectale* to produce butyrate, and butyrate is known for its anti-inflammatory properties, its role in intestinal cell development and gene expression, and its protective effect against colon rectal cancer and colitis [66]. Detailed overviews on the dynamics involved in the production and absorption of acetate, propionate and butyrate within the human gut and on the functional role of SCFA have been recently provided by Facchin et al. [67] and Ravagan and Hemalatha [65]. 

The best way to administer SCFA is to introduce food matrices containing their precursors such as dietary fibers. In this context, the functional foods enriched with prebiotic fiber can considerably contribute to increase the amount of SCFA in the human gut. Recently, the positive effects of inulin and relevant compounds have been demonstrated in the prevention and management of a wide range of diseases. A very recent study reported the efficacy of inulin and derived SCFA in decreasing the expression of genes linked to fibrosis and collagen production in mouse embryonic fibroblast cell line NIH/3T3 in a mouse model of chronic radiation enteropathy [10], thus suggesting a novel application for the inulin-enriched matrices in chronic radiation-induced colonic fibrosis. Interestingly, van Trijp et al. [68] studied the acute fermentation kinetics of GOS and chicory FOS in the (small) intestine in humans using a naso-intestinal catheter and the fate of SCFA as substrates for glucose and lipid metabolism by the host after infusion of ^13^C-SCFA to understand the mechanism by which these metabolites regulate host metabolism. Authors observed that, independently from the delivery location, SCFAs were rapidly absorbed at the delivery site (distal ileum, proximal colon or distal colon).

**Figure 3:**
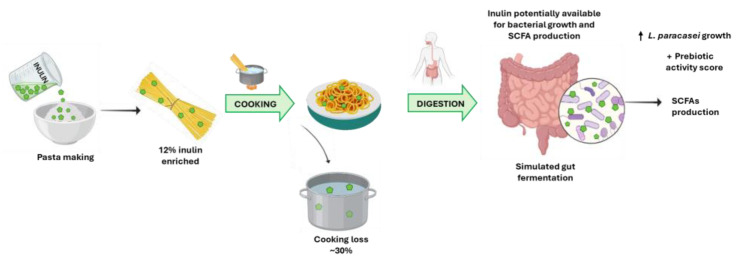
Potential mechanism of action of inulin-enriched pasta and prebiotic effect.

Results from the current study allowed one to support the beneficial health effects that can be related to the consumption of pasta enriched with 12% chicory (low DP) inulin. This percentage of fortification was positively accepted by panelists and was associated with the higher prebiotic activity score and SCFA production higher than CTRL pasta (Figure 3).

## 4. Conclusions

The incorporation of 12% chicory inulin in pasta formulation significantly affected some organoleptic traits, mainly with respect to firmness and bulkiness, and the cooking quality of the final product, and was in any case higher than the threshold of acceptability with respect to the CTRL pasta. Although the cooking process caused a 30% loss of inulin content, the high inulin content allowed one to observe, after simulated gastrointestinal digestion and gut fermentation, a potential prebiotic ability of the innovative pasta. The probiotic strain *Lacticaseibacillus paracasei* IMPC2.1 showed its ability to grow in digested inulin-enriched pasta with the SCFA production to a higher extent than in the CTRL pasta and at the same level as in the presence of glucose or FOS. Further studies will be necessary to evaluate the influence of the degree of polymerization of inulin on gut microbiota modulation, thus improving intestinal health benefits.

## Figures and Tables

**Table 1:** Microorganisms used for the preliminary prebiotic evaluation of chicory inulin.

Microorganism	Origin	Media Incubation Conditions
*Bifidobacterium animalis* ssp. *lactis* Bb12^®^	Chr. Hansen (Hørsholm, Denmark)	MRS Supplemented with 0.05% (*w*/*v*) L-cysteine-HCl Anaerobic, 37 °C
*Lacticaseibacillus casei* 01	Chr. Hansen (Hørsholm, Denmark)	MRS, aerobic 37 °C
*Lacticaseibacillus rhamnosus* GG	Chr. Hansen (Hørsholm, Denmark)	MRS, aerobic 37 °C
*Lacticaseibacillus casei* 431	Chr. Hansen (Hørsholm, Denmark)	MRS, aerobic 37 °C
*Lacticaseibacillus paracasei* IMPC2.1	CNR-ISPA	MRS, aerobic 37 °C
*Lacticaseibacillus casei* IMPC4.1	CNR-ISPA	MRS, aerobic 37 °C
*Lacticaseibacillus paracasei/casei* P1	CNR-ISPA	MRS, aerobic 37 °C

**Table 2:** Quality traits of cooked spaghetti samples.

Trait	CTRL	Inulin Fortified Pasta	*p* ^1^ (0.05)
*Sensory properties*			
Elasticity	6.5 ± 0.2	6.5 ± 0.3	ns
Firmness	7.3 ± 0.4	6.3 ± 0.4	***
Fibrous	6.3 ± 0.3	6.2 ± 0.3	ns
Bulkiness	7.8 ± 0.2	6.0 ± 0.2	***
Adhesiveness	6.5 ± 0.3	6.0 ± 0.1	*
Color	8.0 ± 0.1	7.5 ± 0.1	*
Odor	8.0 ± 0.1	7.0 ± 0.1	**
Taste	7.8 ± 0.2	7.0 ± 0.1	*
Overall Quality Score	7.3 ± 0.3	6.6 ± 0.1	*
*Color*			
L*	55 ± 3.0	66 ± 2.0	***
a*	5.5 ± 1.2	3.1 ± 0.8	***
b*	17 ± 1.8	10 ± 2.1	***
*Cooking quality*			
OCT (min)	11.5	7.5	***
Swelling index	2.20 ± 0.06	1.79 ± 0.04	*
Water Absorption (%)	89 ± 1.5	96 ± 0.7	***

^1^ ***, ** and * indicate significant differences at *p* < 0.001, *p* < 0.01 and *p* < 0.05, respectively; “ns” means not significant.

**Table 3:** Individual and Total Amino Acid (TAA) content (g 100 g^−1^, ±SD) in pasta (CTRL) and inulin fortified pasta.

Amino Acids	CTRL	Inulin-Fortified Pasta	*p* ^1^ Value
	g/100 g		
Alanine	2.369 ± 0.005	2.153 ± 0.011	***
Leucine	1.446 ± 0.008	1.530 ± 0.002	***
Methionine	0.247 ± 0.004	0.259 ± 0.003	ns
Phenylalanine	0.016 ± 0.002	0.029 ± 0.004	ns
Lysine	1.013 ± 0.002	0.971 ± 0.004	***
Cystine	1.517 ± 0.023	1.526 ± 0.004	ns
Hystidine	1.831 ± 0.004	1.813 ± 0.003	*
TAA	8.4365 ± 0.012	8.2795 ± 0.013	***

^1^ *** and * indicate significant differences at *p* < 0.001, and *p* < 0.05, respectively; “ns” means not significant.

**Table 4:** Growth kinetic data expressed as induction times (lag phase duration) calculated for seven bacterial strains grown in MRS broth enriched with selected carbon source (inulin from chicory, In; fructore, FR; fructo-oligosaccharides, FOSs) or without carbon source (control) and monitored in the BioScreen C system until stationary phase. Additionally, the maximum optical density (600 nm) registered during the 36 h of incubation is also shown as a measure of maximum bacterial growth ability in the presence of the selected carbon source.

	Maximum OD in 36 h						
Prebiotic Compound	*L. casei* 431	*L. casei* 01	*L. paracasei* IMPC2.1	*L. paracasei* IMPC4.1	*L. rhamnosus* GG	*B. animalis* subsp. *lactis Bb*12	*L. paracasei/casei* P1
In	1.18 ± 0.008 ^Ab^	0.44 ± 0.036 ^Aa^	1.41 ± 0.010 ^Ac^	1.48 ± 0.009 ^Ad^	0.41 ± 0.035 ^Aa^	1.23 ± 0.040 ^Ab^	0.46 ± 0.008 ^Aa^
FR	1.20 ± 0.022 ^Ac^	1.57 ± 0.010 ^Ba^	1.49 ± 0.014 ^Bb^	1.47 ± 0.007 ^Ab^	1.55 ± 0.006 ^Ba^	1.15 ± 0.003 ^Bd^	1.15 ± 0.025 ^Bd^
FOS	1.19 ± 0.06 ^Ac^	0.43 ± 0.012 ^Aa^	1.39 ± 0.012 ^Ad^	1.48 ± 0.012 ^Ae^	0.44 ± 0.003 ^Aa^	1.19 ± 0.004 ^ABc^	0.47 ± 0.009 ^Ab^
Control	0.19 ± 0.003 ^Ba^	0.36 ± 0.003 ^Cde^	0.35 ± 0.001 ^Cd^	0.37 ± 0.001 ^Be^	0.35 ± 0.005 ^Cd^	0.24 ± 0.005 ^Cb^	0.30 ± 0.001 ^Cc^
	Induction period (h)						
In	7	– ^1^	3.5	3.5	–	4	–
FR	3	4	3	3	6	1.5	3
FOS	4	–	3.5	3.5	–	2	–
Control	–	–	–	–	–	–	–

One-way ANOVA and Tukey tests were applied to determine the significant differences (*p* < 0.05) between different carbon sources (column, capital letters) for each strain and among strains (row, lowercase letter). ^1^ “–” means that no growth was observed and the induction periods were not calculated.

**Table 5:** Changes in the bacterial populations (log_10_ CFU/mL) in in vitro fermentation at T0 (inoculum) and 48 h using pasta enriched with inulin as carbohydrate source in comparison to glucose and FOS. As additional control, pasta without added carbohydrate sources was added (CTRL).

Sample	T0	T48	T0	T48	
	**Mono-Cultures**				
	***L. paracasei* IMPC2.1**		***E. coli* ATCC35401**		**Prebiotic Activity Score_Monoculture**
Inulin-enriched pasta	6.81 ± 0.68 ^a^	9.00 ± 0.79 ^c^	5.75 ± 0.50 ^a^	7.63 ± 1.37 ^c^	0.66 ± 0.17 ^a^
FOS	7.07 ± 0.18 ^a^	8.51 ± 0.16 ^bc^	5.88 ± 0.39 ^a^	7.16 ± 0.28 ^bc^	0.35 ± 0.21 ^a^
Glucose	7.00 ± 0.14 ^a^	8.63 ± 1.01 ^bc^	5.92 ± 0.71 ^ab^	7.81 ± 0.31 ^c^	-
CTRL pasta	7.07 ± 0.06 ^a^	7.81 ± 0.53 ^ab^	5.55 ± 0.23 ^a^	8.08 ± 1.21 ^c^	−0.88 ± 0.39 ^b^
	**Co-Cultures**				
	***L. paracasei* IMPC2.1**		***E. coli* ATCC35401**		**Prebiotic Activity Score_Co-Culture**
Inulin-enriched pasta	6.86 ± 0.89 ^ab^	7.82 ± 1.27 ^abc^	5.86 ± 0.84 ^a^	6.06 ± 0.08 ^a^	0.08 ± 0.03 ^ab^
FOS	6.61 ± 0.61 ^a^	8.21 ± 0.25 ^bc^	6.00 ± 0.26 ^a^	5.37 ± 0.17 ^a^	0.32 ± 0.16 ^b^
Glucose	6.59 ± 0.58 ^a^	9.09 ± 0.13 ^c^	5.86 ± 0.60 ^a^	5.37 ± 0.31 ^a^	0.39 ± 0.25 ^b^
CTRL pasta	6.81 ± 0.43 ^a^	7.31 ± 0.16 ^ab^	5.50 ± 0.29 ^a^	7.36 ± 0.19 ^b^	−0.22 ± 0.38 ^a^

For each strain, two factor ANOVA and Fisher tests were applied to determine the significant differences (*p* < 0.05), denoted by corresponding lowercase letters. Significant differences (*p* < 0.05) between the prebiotic indices were indicated by corresponding lowercase letters.

**Table 6:** SCFA and lactate production of *L. paracasei* IMPC2.1 in the presence of in vitro digested pasta after 48 h fermentation.

Sample	Lactate mMol/L	Acetate mMol/L	Butyrate mMol/L	Valerate mMol/L	Propionate mMol/L
	*L. paracasei* IMPC2.1				
Inulin-enriched pasta	26.25 ± 2.09 ^ab^	4.78 ± 0.38 ^de^	-	3.05 ± 0.58 ^e^	4.92 ± 1.83 ^bcd^
FOS	51.16 ± 9.44 ^cd^	3.12 ± 0.91 ^c^	10.75 ± 1.81 ^b^	2.92 ± 0.40 ^e^	5.40 ± 0.41 ^d^
Glucose	65.51 ± 18.39 ^d^	4.10 ± 1.18 ^cd^	6.00 ± 0.68 ^a^	2.59 ± 0.69 ^de^	4.95 ± 1.64 ^cd^
CTRL pasta	9.18 ± 0.37 ^a^	-	-	0.98 ± 0.09 ^bc^	2.30 ± 0.12 ^a^
	*E. coli* ATCC35401				
Inulin-enriched pasta	6.25 ± 0.35 ^a^	0.86 ± 0.23 ^ab^	17.69 ± 4.70 ^d^	2.93 ± 0.64 ^e^	1.53 ± 1.25 ^a^
FOS	30.79 ± 7.87 ^abc^	1.63 ± 0.54 ^ab^	8.84 ± 0.10 ^ab^	2.37 ± 0.14 ^de^	2.69 ± 1.69 ^ab^
Glucose	13.79 ± 8.53 ^a^	0.69 ± 0.15 ^a^	8.75 ± 1.37 ^ab^	2.69 ± 0.28 ^de^	1.81 ± 0.60 ^a^
CTRL pasta	5.50 ± 0.70 ^a^	0.84 ± 0.21 ^ab^	12.18 ± 3.09 ^bc^	2.24 ± 0.33 ^de^	1.07 ± 0.60 ^a^
	Co-cultures				
Inulin-enriched pasta	10.84 ± 7.98 ^a^	0.89 ± 0.31 ^ab^	15.87 ± 0.81 ^cd^	2.40 ± 0.59 ^de^	2.38 ± 0.13 ^a^
FOS	46.77 ± 33.69 ^bcd^	1.93 ± 0.41 ^b^	8.18 ± 1.02 ^ab^	1.86 ± 0.51 ^cd^	3.45 ± 0.42 ^abcd^
Glucose	137.31 ± 0.20 ^e^	0.92 ± 0.09 ^ab^	9.99 ± 1.37 ^ab^	-	3.01 ± 2.01 ^abc^
CTRL pasta	8.08 ± 0.40 ^a^	-	-	0.50 ± 0.14 ^ab^	2.36 ± 0.47 ^a^

For each acid, one-way ANOVA and Fisher tests were applied to determine the significant differences (*p* < 0.05) denoted by corresponding lowercase letters.

## Data Availability

The original contributions presented in the study are included in the article, further inquiries can be directed to the corresponding authors.
